# Sitting patterns in cardiovascular disease patients compared with healthy controls and impact of cardiac rehabilitation

**DOI:** 10.1111/sms.14202

**Published:** 2022-06-18

**Authors:** Pam ten Broeke, Bram M. A. van Bakel, Esmée A. Bakker, Debby G. J. Beckers, Sabine A. E. Geurts, Dick H. J. Thijssen, Thijs M. H. Eijsvogels, Erik Bijleveld

**Affiliations:** ^1^ Behavioural Science Institute Radboud University Nijmegen The Netherlands; ^2^ Radboud Institute for Health Sciences, Department of Physiology Radboud University Medical Center Nijmegen The Netherlands; ^3^ Research Institute for Sport and Exercise Sciences Liverpool John Moores University Liverpool UK

**Keywords:** accumulation, circadian, sedentary behavior, survival analysis, time‐to‐event analysis

## Abstract

**Purpose:**

To identify how and when to intervene in cardiovascular disease (CVD) patients' sedentary behavior, we moved beyond studying total volume of sitting and examined sitting patterns. By analyzing the timing of stand‐to‐sit and sit‐to‐stand transitions, we compared sitting patterns (a) between CVD patients and healthy controls, and (b) before and after cardiac rehabilitation (CR).

**Methods:**

One hundered twenty nine CVD patients and 117 age‐matched healthy controls continuously wore a tri‐axial thigh‐worn accelerometer for 8 days (>120 000 posture transitions). CVD patients additionally wore the accelerometer directly and 2 months after CR.

**Results:**

With later time of the day, both CVD patients and healthy controls sat down sooner (i.e., shorter standing episode before sitting down; HR = 1.01, 95% CI [1.011, 1.015]) and remained seated longer (HR = 0.97, CI [0.966, 0.970]). After more previous physical activity, both groups sat down later (HR = 0.97, CI [0.959, 0.977]), and patients remained seated longer (HR = 0.96; CI [0.950, 0.974]). Immediately and 2‐months following CR, patients sat down later (HR_post‐CR_ = 0.96, CI [0.945, 0.974]; HR_follow‐up_ = 0.96, CI [0.948, 0.977]) and stood up sooner (HR_post‐CR_ = 1.04, CI [1.020, 1.051]; HR_follow‐up_ = 1.03, CI [1.018, 1.050]). These effects were less pronounced with older age, higher BMI, lower sedentary behavior levels, and/or higher physical activity levels at baseline.

**Conclusion:**

Cardiac rehabilitation programs could be optimized by targeting CVD patients' sit‐to‐stand transitions, by focusing on high‐risk moments for prolonged sitting (i.e., in evenings and after higher‐than‐usual physical activity) and attending to the needs of specific patient subgroups.

## 1 INTRODUCTION

Patients with cardiovascular disease (CVD) spend around 10 h of their daily waking time sitting.^1^, ^2^ High levels of sedentary behavior, particularly in prolonged, uninterrupted periods of time, are associated with an increased risk of cardiovascular morbidity and mortality.^3^, ^4^, ^5^, ^6^ Therefore, sedentary behavior likely plays an important role in the prognosis of CVD patients, and this patient group should benefit from adopting a more active lifestyle.

Prior research has mainly focused on the *volume* of sedentary behavior amongst CVD patients: CVD patients spend approximately 1 h a day more in a sedentary posture compared with healthy individuals.^2^ Since only a few studies have explored CVD patients' sitting patterns,^5^ we know little about how and when CVD patients accumulate these volumes of sedentary behavior. Detailed insight into the duration of CVD patients' sitting and standing episodes and how these are distributed throughout the day, is crucial to identify how and when to effectively intervene in CVD patients' sedentary behavior.

**FIGURE 1:**
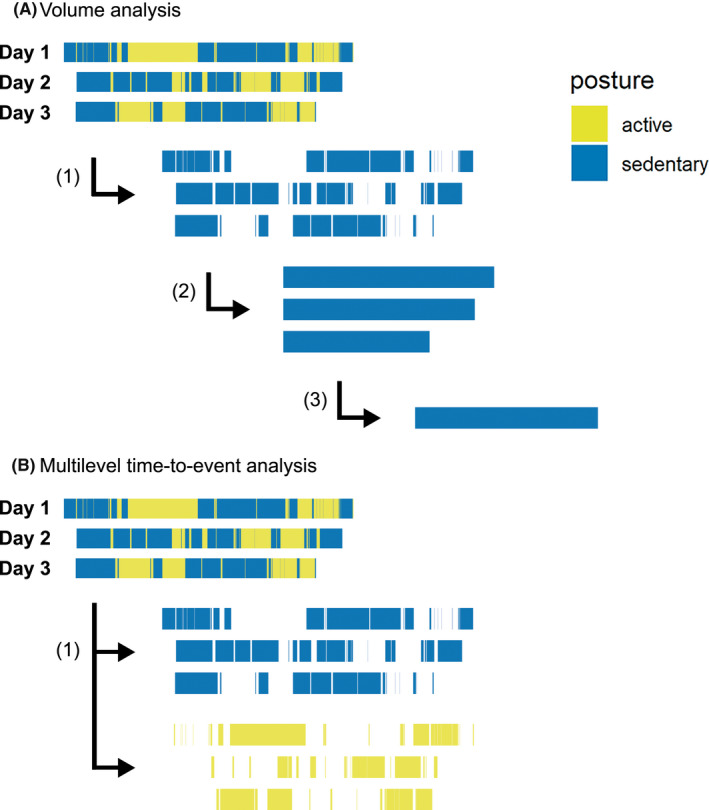
Visualization of the differences between volume analysis and multilevel time‐to‐event analysis to analyze the sedentary behavior of an individual on three waking days. (A) In volume analysis, (1) all sedentary episodes are extracted from the raw accelerometry data, (2) sedentary episodes are summed into total sedentary time per day, and (3) then further summarized as the average sedentary time per person, which is the unit of analysis. Relevant information about sitting patterns is lost during pre‐processing. (B) In multilevel time‐to‐event analysis, (1) all sedentary episodes *and* all active episodes are extracted from the data separately, and then analyzed without further pre‐processing, making it possible to examine sitting and standing patterns in detail^7^

In this study, we examined patterns of sedentary behavior in CVD patients and age‐matched healthy controls. Specifically, by adopting a recently introduced analytical approach,^7^ we analyzed the timing of stand‐to‐sit transitions (i.e., how long participants stand before sitting down) and the timing of sit‐to‐stand transitions (i.e., how long participants sit before standing up; Figure 1). In previous research, this pattern analysis of office workers' sedentary behavior provided novel insights into how office workers regulate their posture transitions throughout the day. We hypothesized that CVD patients generally sit down sooner (i.e., shorter standing episode before sitting down) and remain seated longer (i.e., longer sitting episode before standing up), compared with healthy controls. Moreover, we expected that CVD patients and healthy controls distribute the timing of their stand‐to‐sit and sit‐to‐stand transitions differently throughout the day, as well as in relation to previous physical activity.

Contemporary cardiac rehabilitation programs have a multidisciplinary approach (including exercise training, psychosocial management, and nutrition counseling). However, most programs lack components that specifically target CVD patients' sedentary behavior. Nevertheless, a recent study shows that CVD patients engage in slightly less total sedentary time (~0.4 h) after participating in such a program.^2^ A more detailed understanding of this change in terms of sitting patterns could help to better understand the impact of CR on prolonged sitting, and to identify how to optimize cardiac rehabilitation programs in terms of the prevention of prolonged sitting.

Therefore, using the same novel analytical approach, we also assessed changes in CVD patients' sitting patterns from before to after a contemporary cardiac rehabilitation program. We hypothesized that after the cardiac rehabilitation program, CVD patients sit down later (i.e., longer standing episode before sitting down) and stand up sooner (i.e., shorter sitting episode before standing up). In addition, we explored whether these changes in sitting patterns differed based on patient‐ and disease characteristics.

## 2 METHODS

We used data from a recent study that compared volumes of sedentary behavior and physical activity between CVD patients and healthy controls and assessed the impact of cardiac rehabilitation on sedentary behavior and physical activity.^2^ We preregistered our research questions, hypotheses, data‐processing steps, and analyses at the open science framework (URL to the preregistration).

### 2.1 Participants and design

One hundred twenty nine CVD patients were recruited at the start of participating in a contemporary cardiac rehabilitation (CR) program. In addition, 117 age‐matched participants without established CVD and without a diagnosis of hypertension, diabetes mellitus, or dyslipidemia were recruited as healthy controls via social media advertisement and via friends and family of the CVD patients. For the first aim of the study, all participants underwent 8 days of sedentary behavior data assessment. For the second aim of the study, 8 days of sedentary behavior data of CVD patients was also assessed directly post‐CR, and at 2 months follow‐up. For the post‐CR measure, we included data from 112 CVD patients in our analysis (8 patients had discontinued the CR program, 5 dropped out of the study; 4 experienced technical problems). For the follow‐up measure, we included data from 110 patients (2 additional patients dropped out of the study; 4 experienced technical problems). Patients who dropped out and/or were excluded due to technical problems, did not significantly differ from patients who were included in the analyses on age, BMI, and average MVPA level (all *p*s > 0.07), but showed significantly higher average sitting levels at baseline compared to patients who were included in the analyses (post‐CR: *M*_included_ = 10.1, SD_included_ = 1.5, *M*_excluded_ = 10.9, SD_excluded_ = 1.6, *p* = 0.04; follow‐up: *M*_included_ = 10.0, SD_included_ = 1.5, *M*_excluded_ = 11.2, SD_excluded_ = 1.6, *p* = 0.002). The study procedure was approved by the medical ethical committee of the Radboud University Medical Centre (#2017–3315). All participants provided written informed consent.

### 2.2 Procedure

Participants completed a questionnaire on demographics (age, sex, education level, marital status, and employment status). For CVD patients, additional patient‐ and disease‐characteristics were retrieved from electronic patient files. Sedentary behavior was recorded using an activPAL3 micro monitor (PAL Technologies). Participants were instructed to continuously wear the monitor for 8 consecutive days, and to record wake and sleep times in a diary.

All CVD patients underwent the CR program, which included a 6‐week exercise program of two 1‐h exercise sessions per week. Based on individual patient needs, the program was supplemented with additional modules (e.g., mental health and stress relief, social health, and/or cardiovascular risk management).

## 3 MEASURES

### 3.1 Patient‐ and disease characteristics

Characteristics included age (in years), sex, Body Mass Index (BMI; kg/m^2^), average daily sitting level (in hours per day), average daily moderate‐to‐vigorous physical activity (MVPA) level (in minutes per day), type of CVD (Acute Coronary Syndrome and angina pectoris versus other [congenital heart disease; heart failure; heart rhythm disorder; heart valve disease; other]), polypharmacy (>5 types of medication), treatment (Coronary Artery Bypass Grafting versus other [electro cardioversion/ablation/mini‐maze procedure; heart valve replacement, pacemaker or ICD implementation, PCI, and medication only]), diabetes mellitus (yes/no), dyslipidemia (yes/no), arthrosis (yes/no), rheumatoid arthritis (yes/no), alcohol consumption (high [>14 alcoholic drinks per week for men and >7 for women] versus low), and smoking (currently smoking versus not currently smoking).* For CVD patients, we had 21 missing values on alcohol use. For healthy controls, we had 24 missing values on alcohol use and one on smoking.

### 3.2 Sedentary behavior and physical activity

For the assessment of sedentary behavior, participants wore a waterproofed activPAL monitor^8^ on their upper‐right thigh. Self‐reported sleep/wake‐times (using a 40‐min time‐window around the self‐reported times) in combination with a modified version of a previously developed algorithm^9^ were used to identify and exclude activPAL data during sleep and non‐wear. Specifically, the algorithm identifies each sitting, standing, or stepping episode as valid data versus sleep/non‐wear data (e.g., long periods without posture change). In addition, the algorithm determines whether each measurement day constitutes a valid day or an invalid day. A measurement day is considered invalid when (a) one activity takes up more than 95% of total awake time, (b) the number steps is below 1000, or (c) the number of hours awake is less than 10.

From the activPAL data of the baseline measurement, we calculated for each participant the characteristics average daily sitting level as the average daily total sitting time (in hours), and average daily MVPA level as the average daily time (in minutes) spent stepping with MET (metabolic equivalent) values ≥3.

### 3.3 Time of the day and activity in the preceding 5 h

Time of the day (in hours) was calculated from the activPAL data in hours since midnight. Activity in the preceding 5 h (in hours) was calculated from the activPAL data as the total time the participant spent active (all non‐sitting behavior, including both light physical activity [LIPA] and MVPA) in the 5 h prior to each stand‐to‐sit or sit‐to‐stand transition. We chose a 5‐h time window based on previous studies on muscle fatigue showing that physical discomfort tends to set in within 2–5 h of activity.^10^, ^11^

### 3.4 Data‐analysis

To test the difference in average daily sitting time at baseline between patients and healthy controls, we fitted a linear regression model with average daily sitting time (in hours) as dependent variable, and group (CVD vs. control) as predictor. To test the difference in average daily sitting time in CVD patients from before to after CR, we fitted a mixed‐effects model with average daily sitting time (in hours) as dependent variable, measurement moment (pre‐CR; post‐CR; follow‐up) as predictor, and a random intercept for participants.

We modeled the timing of sit‐to‐stand and stand‐to‐sit transitions using multilevel time‐to‐event analysis,^7^ also known as multilevel survival analysis.^12^, ^13^ Our event of interest was sit‐to‐stand transitions and stand‐to‐sit transitions. We fitted several Cox regression models with as dependent variable the *hazard* (i.e., the probability that an event occurs per unit of time, given that the event has not happened yet) of sitting down when standing, and the hazard to standing up when sitting. As predictors, we included group (CVD vs. control), time of the day, activity in the preceding 5 h, measurement moment (pre‐CR; post‐CR; follow‐up), or a patient‐ and disease‐characteristics predictor. A multilevel framework was used, because each individual engages in multiple stand‐to‐sit and sit‐to‐stand (i.e., events nested within individuals). Specifically, in each Cox regression model, we included a *frailty term* for participant, accounting for the random variability in baseline hazard between individuals (comparable with a random intercept in linear mixed‐level models).

For Aim 1 (i.e., compare CVD patients and healthy controls, and the relation with time of the day, and activity in the preceding 5 h), we used the pre‐CR data of the CVD patients and the data of the healthy controls (i.e., mixed between‐ and within‐subjects). For Aim 2 (i.e., changes from before to after cardiac rehabilitation), we used the pre‐CR, post‐CR, and follow‐up data of the CVD patients (i.e., within‐subjects). To interpret significant associations, we used model estimations to calculate *P*(time‐to‐event > 15 min) for stand‐to‐sit transitions and *P*(time‐to‐event > 30 min) for sit‐to‐stand transitions, for two values of time of the day (i.e., 8 a.m. vs. 8 p.m.; we chose these values to illustrate the difference between mornings and evenings) or two values of relative (i.e., within‐person) activity in the preceding 5 h (i.e., 10th percentile [lower‐than‐usual] vs. 90th percentile [higher‐than‐usual]). The choice of 15 and 30 min as meaningful values for relatively long standing and sitting episodes was based on a combination of previous work^14^ and the distribution of episode duration in our data (>10% of sitting or standing episodes above the cut‐off). Details regarding data preparation and model fitting can be found in the Appendix S1.

## 4 RESULTS

### 4.1 Participant characteristics and descriptives

Table 1 provides an overview of baseline characteristics. CVD patients had an average age of 62 (SD = 10) and an average BMI of 27.5 (SD = 4.42), compared to 60 (SD = 9) and 24.6 (SD = 3.42) in healthy controls. 74% of CVD patients were male and 72% were unemployed, compared to 62% and 28% in healthy controls.

**TABLE 1:** Baseline characteristics of CVD patients (*N* = 129) and healthy controls (*N* = 117)

Characteristic	*n* (%) / mean (SD)	
	Patients	Healthy controls
Age (in years)	62 (10)	60 (9)
Sex		
Female	33 (26%)	44 (38%)
Male	96 (74%)	73 (62%)
BMI (kg/m^2^)	27.5 (4.42)	24.6 (3.42)
Current employment status		
Employed	36 (28%)	73 (62%)
Unemployed	93 (72%)	44 (38%)
Average sitting level (hours per day)	10.4 (1.5)	9.2 (1.5)
Average MVPA level (minutes per day)	40.5 (18.1)	61.2 (25.1)
Type of CVD		
Acute Coronary Syndrome (ACS) and angina pectoris	95 (74%)	‐
Other (congenital heart disease; heart failure; heart rhythm disorder; heart valve disease; other)	34 (26%)	‐
Polypharmacy (>5 types of medication)	18 (14%)	‐
Treatment		
Coronary Artery Bypass Grafting (CABG)	30 (23%)	‐
Other (electro cardioversion / ablation / mini‐maze procedure; heart valve replacement, pacemaker or ICD implementation, PCI, and medication only)	99 (77%)	‐
Diabetes mellitus	22 (17%)	‐
Dyslipidemia	45 (35%)	‐
Arthrosis	6 (5%)	‐
Rheumatoid arthritis	7 (5%)	‐
Alcohol consumption		
High (>14 alcoholic drinks per week for men and >7 for women)	9 (7%)	9 (8%)
Low (≤14 alcoholic drinks per week for men and ≤7 for women)	99 (77%)	84 (72%)
Smoking status		
Currently smoking	9 (7%)	9 (8%)
Not currently smoking	120 (93%)	107 (91%)

In total, we had data on 37 713 stand‐to‐sit and 37 009 sit‐to‐stand transitions for CVD patients at baseline, 34 045 stand‐to‐sit and 33 413 sit‐to‐stand transitions at post‐CR, and 34 937 stand‐to‐sit and 34 292 sit‐to‐stand transitions at follow‐up. In addition, we had 39 951 stand‐to‐sit and 39 240 sit‐to‐stand transitions for healthy controls.

### 4.2 Average daily sitting time

Cardiovascular disease patients spent significantly more time sedentary at baseline (*M* = 10.4, SD = 1.5) compared with healthy controls (*M* = 9.2, SD = 1.5; *b* = 1.25, *p* < 0.001).† Compared with before CR (*M* = 10.4, SD = 1.5), CVD patients spent significantly less time sedentary directly after CR (*M* = 9.9, SD = 1.5; *p* < 0.001) and at follow‐up (*M* = 9.9, SD = 1.6; *p* < 0.01).

### 4.3 Sitting patterns of CVD patients and healthy controls

Cardiovascular disease patients and healthy controls did not significantly differ in the overall timing of their stand‐to‐sit transitions and sit‐to‐stand transitions (Tables 2 and 3). Nonetheless, the probabilities were in the expected direction: The probability of a standing episode >15 min was 10% in CVD patients and 13% in healthy controls. The probability of a sitting episode >30 min was 12% for CVD patients and 9% for healthy controls.

**TABLE 2:** Results of the shared frailty Cox regression models for group, time of the day, and activity in the preceding 5 h on the hazard of sitting down when standing

Predictor	Estimate	df	SE	HR	HR 95% CI
Group—hazard of sitting down when standing					
Random effect *θ*	0.358***	242.8			
Group^a^	0.129	1	0.072	1.14	[0.988; 1.311]
Time of the day, group, and interaction – hazard of sitting down when standing					
Random effect θ	0.366***	242.9			
Time of the day	0.013***	1	0.001	1.01	[1.011; 1.015]
Group^a^	0.034	1	0.036	1.04	[0.893; 1.200]
Time of the day x Group^a^	0.007***	1	0.002	1.01	[1.004; 1.010]
Simple slope: CVD patients	0.020***	1	0.001	1.02	[1.018; 1.022]
Simple slope: Controls	0.013***	1	0.001	1.01	[1.011; 1.015]
Activity in the preceding 5 h, group, and interaction—hazard of sitting down when standing					
Random effect *θ*	0.295***	242.3			
Activity in the preceding 5 h	−0.033***	1	0.005	0.97	[0.959; 0.977]
Group^a^	0.126	1	0.024	1.13	[0.995; 1.294]
Activity in the preceding 5 h x Group^a^	−0.008	1	0.008	0.99	[0.978; 1.008]

Abbreviations: CI, confidence interval; df, degrees of freedom; HR, hazard ratio; SE, standard error.

*** *p* < 0.001.

^a^ Group was coded as 0 = control vs. 1 = CVD patients.

**TABLE 3:** Results of the shared frailty Cox regression models for group, time of the day, and activity in the preceding 5 h on the hazard of standing up when sitting

Predictor	Estimate	df	SE	HR	HR 95% CI
Group – hazard of standing up when sitting					
Random effect θ	0.314***	242.1			
Group^a^	−0.145	1	0.081	0.87	[0.738; 1.014]
Time of the day, group, and interaction – hazard of standing up when sitting					
Random effect θ	0.334***	242.3			
Time of the day	−0.032***	1	0.001	0.97	[0.966; 0.970]
Group^a^	−0.162	1	0.086	0.85	[0.719; 1.006]
Time of the day x Group^a^	0.001	1	0.002	1.001	[0.998; 1.004]
Activity in the preceding 5 h, group, and interaction – hazard of standing up when sitting					
Random effect θ	0.312***	242.1			
Activity in the preceding 5 h	−0.009	1	0.005	0.99	[0.981; 1.001]
Group^a^	−0.104	1	0.082	0.90	[0.767; 1.058]
Activity in the preceding 5 h x Group^a^	−0.030***	1	0.008	0.97	[0.955; 0.987]
Simple slope: CVD Patients	−0.039***	1	0.006	0.96	[0.950; 0.974]
Simple slope: Controls	−0.009	1	0.005	0.99	[0.981; 1.001]

Abbreviations: CI, confidence interval; df, degrees of freedom; HR, hazard ratio; SE, standard error.

*** *p* < 0.001.

^a^ Group was coded as 0 = control vs. 1 = CVD patient.

**FIGURE 2:**
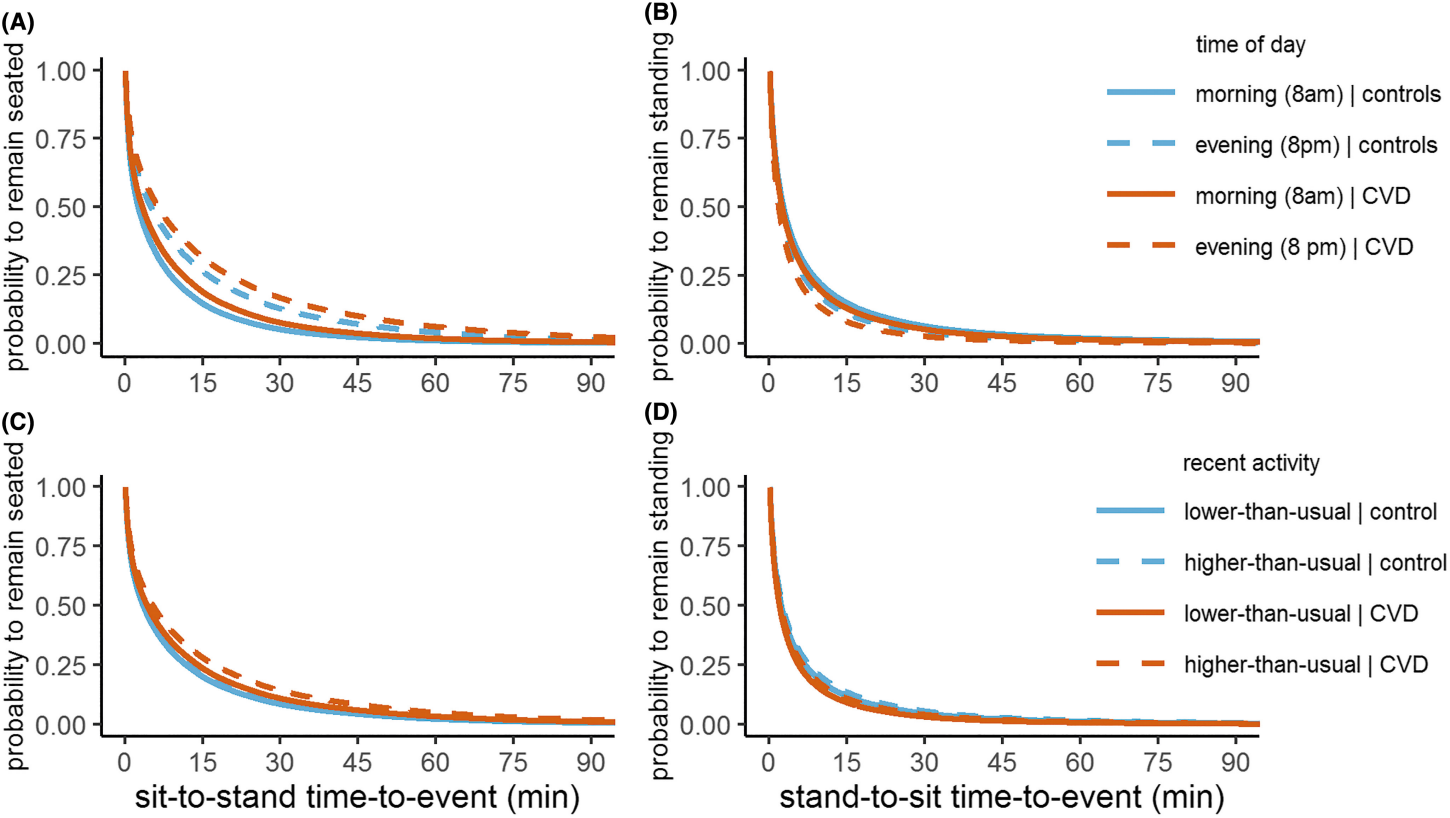
Estimated probability to remain seated over the duration of a sitting episode (sit‐to‐stand time‐to‐event; panel A and C) or remain standing over the duration of a standing episode (stand‐to‐sit time‐to‐event; Panel B and D) in CVD patients (*N* = 129) and healthy controls (*N* = 117) for prototypical values of time of the day (i.e., 8 a.m. vs. 8 p.m.; Panel A and B) or prototypical values of relative (i.e., within‐person) activity in the preceding 5 h (i.e., 10th percentile [lower‐than‐usual] vs. 90th percentile [higher‐than‐usual]; Panel C and D). (A) Participants were more likely to remain seated for longer durations in evenings. (B) Participants were less likely to remain standing for longer durations in evenings. (C) CVD patients were more likely to remain seated for longer durations after they were more active than usual in the preceding 5 h. (D) Participants were more likely to remain standing for longer durations when they were more active than usual in the preceding 5 h

Time of the day significantly predicted the timing of sitting down when standing, qualified by a significant interaction with group (Table 2 and Figure 2). Specifically, both CVD patients and healthy controls sat down sooner later on the day compared with earlier on the day, but the effect was stronger in CVD patients (as indicated by the significant interaction). To illustrate, for CVD patients, the probability of a standing episode >15 min was 13% in mornings (8 a.m.) and 8% in evenings (8 p.m.). For healthy controls, the probability of a standing episode >15 min was 15% in mornings (8 a.m.) and 11% in evenings (8 p.m.).

Time of the day also significantly predicted the timing of standing up when sitting, but the interaction with group was not significant (Table 3 and Figure 2). Specifically, both CVD patients and healthy controls remained seated longer later on the day compared with earlier on the day. This effect did not differ between CVD patients and healthy controls (as indicated by the insignificant interaction). To illustrate, for CVD patients, the probability of a sitting episode >30 min was 8% in mornings (8 a.m.) and 17% in evenings (8 p.m.). For healthy controls, the probability of a sitting episode >30 min was 5% in mornings (8 a.m.) and 13% in evenings (8 p.m.). Additional exploratory analyses indicated that these effects of time of the day were stronger for older participants than for younger participants (Table S1).

Activity in the preceding 5 h significantly predicted the timing of sitting down when standing, but the interaction with group was not significant (Table 2 and Figure 2). Specifically, both CVD patients and healthy controls remained standing longer when they had been more physically active in the preceding 5 h. This effect did not differ between CVD patients and healthy controls (as indicate by the insignificant interaction). To illustrate, for CVD patients, the probability of a standing episode >15 min was 9% after lower‐than‐usual activity (10th percentile) and 11% after higher‐than‐usual activity (90th percentile). For healthy controls, the probability of a standing episode >15 min was 12% after lower‐than‐usual activity (10th percentile) and 14% after higher‐than‐usual activity (90th percentile).

Activity in the preceding 5 h did not significantly predict the timing of sitting down when standing, but the interaction was significant (Table 3 and Figure 2). Specifically, CVD patients remained seated longer when they had been more physically active in the preceding 5 h. To illustrate, for CVD patients, the probability of a sitting episodes >30 min was 11% after lower‐than‐usual activity (10th percentile) and 14% after higher‐than‐usual activity (90th percentile). For healthy controls, activity in the preceding 5 h did not significantly predict the timing of sitting down when standing.

### 4.4 Impact of cardiac rehabilitation

The hazard of sitting down when standing changed from baseline to after CR (Table 4). Compared with before CR, CVD patients remained standing slightly longer after CR and at follow‐up. To illustrate the probability of a standing episode >15 min was 9.9% at baseline, 10.5% directly after CR, and 10.7% at follow‐up.

**TABLE 4:** Results of the shared frailty Cox regression models for measurement moment

Predictor	Estimate	df	SE	HR	HR 95% CI
Measurement moment – hazard of sitting down when standing					
Random effect θ	0.954***	128.8			
Measurement moment^a^					
Post‐CR	−0.042***	1	0.008	0.96	[0.945; 0.974]
Follow‐up	−0.039***	1	0.008	0.96	[0.948; 0.977]
Measurement moment—hazard of standing up when sitting					
Random effect θ	0.931***	128.8			
Measurement moment^a^					
Post‐CR	0.035***	1	0.008	1.04	[1.020; 1.051]
Follow‐up	0.033***	1	0.008	1.03	[1.018; 1.050]

Abbreviations: CI, confidence interval; df, degrees of freedom; SE, standard error; HR, hazard ratio.

*** *p* < 0.001.

^a^ Measurement moment was dummy coded with pre‐CR as reference category.

The hazard of standing up when sitting also changed from baseline to after CR (Table 4). Compared with before CR, CVD patients stood up slightly sooner after CR and at follow‐up. To illustrate, the probability of a sitting episode >30 min was 12% at baseline, 11% directly after CR, and 11% at follow‐up.

Models including patient‐ and disease‐characteristics (Figures S1 and S2) suggested that older patients, patients with higher BMI, and patients with higher sitting level at baseline and/or lower MVPA lever at baseline, showed an attenuated change in the timing of sit‐to‐stand and stand‐to‐sit transitions from baseline to after CR.

Secondary analyses indicated that at post‐CR and at follow‐up, sitting patterns in relation to time of the day, and in relation to activity in the preceding 5 h did not differ compared with baseline (Tables S2 and S3).

## 5 DISCUSSION

In this study, we (a) compared CVD patients and age‐matched healthy controls on their sitting patterns throughout the day, and (b) tested changes in CVD patients' sitting patterns after a contemporary cardiac rehabilitation program. In line with previous work,^1^, ^2^ CVD patients engaged in higher volumes of sedentary behavior (1.2 h) compared with healthy controls, and they slightly reduced their sedentary time (with 0.5 h) after engaging in a cardiac rehabilitation program. Extending previous work, we found similarities and differences in how CVD patients and healthy controls accumulated their sedentary time during the day. Both CVD patients and healthy controls sat down sooner and remained seated longer later on the day; the tendency to sit down sooner at the end of the day was slightly stronger in CVD patients than in healthy controls. Furthermore, after engaging in more physical activity within the preceding 5 h, both groups sat down later, but only CVD patients also stood up later. Finally, compared with before participating in a cardiac rehabilitation program, CVD patients remained standing slightly longer and stood up slightly sooner directly after the program, and this change persisted until at least 2‐months after the program. However, these changes were less pronounced in older patients, patients with a higher BMI, and/or patients who were more sedentary and less physically active at baseline.

By moving beyond studying volumes of sedentary behavior, our data shed light on how CVD patients accumulate these volumes of sedentary behavior throughout the day. First, we showed that, like healthy individuals, CVD patients tend to especially engage in prolonged (i.e., unhealthy) sitting episodes in evenings. This pattern may reflect different activities that patients engage in over the time course of a day, such as working, socializing, and relaxing. This pattern may also reflect the fact that humans have an innate tendency to minimize physical effort and to preserve energy,^15^, ^16^ which is even stronger when people feel fatigued compared to when they are well‐rested.^16^ As people tend to feel more fatigued later on the day,^17^ this likely results in a stronger tendency to sit down and remain seated.

Second, unlike healthy individuals, CVD patients are also likely to engage in prolonged sitting episodes after they had engaged in higher‐than‐usual physical activity in the preceding hours. CVD patients are typically less physically fit compared with their healthy peers, for instance in terms of maximal oxygen uptake.^18^ Because of this, they may experience previous physical activity as more strenuous and, as a result, they may experience a stronger subjective need to rest, which in turn prevents them from standing up while sitting.^19^ CVD patients are also more frequently unemployed (71% in our current sample, compared with 38% of healthy controls, see Table 1), and may have different daily routines (e.g., with more opportunities for resting) compared with their healthy peers.

Previous work has already emphasized the need for cardiac rehabilitation programs to pay attention to CVD patients' sedentary behavior.^2^ Our findings provide starting points for *how* and *when* to intervene in sedentary behavior in general, and in CVD patients' sedentary behavior in particular. First, patients showed only minimal changes in their timing of sitting down and standing up after participating in the cardiac rehabilitation program. The effect was plausibly even an overestimation of reality, as patients who dropped out showed higher levels of sedentary behavior at baseline compared to patients who were included (and higher levels of sedentary behavior at baseline were associated with smaller changes in the timing of sitting down and standing up). So, interventions should not only aim to decrease overall sedentary volume, but also aim to change sitting patterns, that is, reduce the number of prolonged, uninterrupted sitting episodes, and stimulate more frequent sit‐to‐stand transitions.^20^, ^21^, ^22^ Increasing sit‐to‐stand transitions may be more realistic and feasible for CVD patients.

Second, while aiming to increase the frequency of sit‐to‐stand transitions, interventions could focus on high‐risk moments for prolonged sitting during the day. Based on our findings, evenings are high‐risk moments for both the general population and CVD patients. For CVD patients, we found that the time immediately after higher‐than‐usual physical activity may also be a high‐risk moment for prolonged sitting. As such, a promising intervention strategy may be to help CVD patients better balance their physical activity and sedentary behavior throughout the day, such that they are less inclined to “rest” with prolonged periods of sitting after having been active for some time. Third, interventions should be sensitive to high‐risk patient groups that may experience more difficulties improving their sitting patterns, but potentially benefit most from sitting less. According to our findings, these are older patients, patients with a higher BMI, and/or patients who were more sedentary and less physically active at baseline.

### 5.1 Strengths and limitations

In this study, we used a recently introduced multilevel time‐to‐event analysis approach^7^ to draw a detailed picture of CVD patients' sitting patterns during the day. This dynamic approach has several advantages over the more traditional approach of only investigating total volume of sedentary behavior. First, our dynamic approach provides a completer and more accurate overview of the natural variability that is characteristic of sedentary behavior. Second, it provides insights into when patients engage in prolonged, uninterrupted periods of sitting, which are especially harmful for cardiovascular health.^5^, ^6^ Third, the dynamic approach is sensitive to also detect minimal changes in CVD patients' sedentary behavior, such as small increases in the time one stands before sitting down. Such minimal changes may be more realistic and feasible for patient groups than reducing daily total sitting time and therefore, may be a more realistic target for interventions. As such, analyzing the timing of stand‐to‐sit and sit‐to‐stand transitions will also be relevant to study sitting patterns in other patient populations for whom sitting and standing behavior is relevant for health, such as adults with metabolic syndrome.^23^

A limitation of the current study is that we could not identify the underlying cause of the differences in daily sitting patterns between CVD patients and healthy controls. Based on theories regarding effort minimization and energy preservation, we could speculate on the potential roles of physical and social differences between patients and healthy individuals. To identify intervention targets, future research should further unravel the environmental, social, and individual determinants and the daily activities that may impact CVD patients' sitting patterns. In particular, in the current study, we did not find evidence that CVD patients' disease characteristics were associated with the timing of stand‐to‐sit and sit‐to‐stand transitions. However, given the exploratory nature of this finding and our broad categorization these characteristics, future studies could aim to further unravel whether and how CVD subtypes, comorbidities, and treatment, and accompanied consequences could impact the way CVD patients accumulate their sedentary behavior throughout the day. Another limitation of the current study was that we did not collect data on the ethnic backgrounds of participants, limiting the generalizability of our findings to varying ethnic populations.

## 6 CONCLUSION

Besides engaging in larger volumes of sedentary behavior, patients with CVD show some differences with the healthy population in how they distribute their sedentary behavior throughout the day. Specifically, patients with CVD tend to engage in prolonged sitting in evenings (just like their healthy peers), but also following previous physical activity (unlike their healthy peers). A contemporary cardiac rehabilitation program that focused on increasing exercise but did not include a focus on sedentary behavior, only slightly reduced prolonged sitting in CVD patients, suggesting that such programs could be optimized by focusing on increasing sit‐to‐stand transitions to break up prolonged sitting more often. Specifically, our data provide insight into high‐risk moments and contexts for prolonged sitting that can be targeted in CVD, such as sitting in evenings and after higher‐than‐usual physical activity. Furthermore, our data suggests that interventions may provide special attention to patients of older age, with a higher BMI, with less sedentary behavior and with more MVPA at baseline, as these patients may experience difficulties to change their sitting patterns.

## 7 PERSPECTIVES

Previous work has emphasized the need for cardiac rehabilitation programs to incorporate a focus on sedentary behavior.^2^ We aimed to shed light on *how* and *when* to intervene in CVD patients' sedentary behavior. In particular, we adopted a recently introduced analytical approach^7^ that moves beyond studying total volume of sitting, to investigate how CVD patients accumulate their sedentary behavior throughout the day. Thereby, we build on prior work that has emphasized the importance of accumulation patterns in the association between sedentary behavior and cardiovascular health.^5^, ^6^, ^24^

We found only a negligible decrease in prolonged sitting after CVD patients engaged in a contemporary cardiac rehabilitation program, suggesting that these programs could be optimized by focusing on increasing sit‐to‐stand transitions to break up prolonged sitting more often. CVD patients especially engaged in prolonged sitting in evenings, and after higher‐than‐usual physical activity, providing valuable target moments for intervention. Changes in prolonged sitting were less pronounced in patients of older age, higher BMI, higher levels of sedentary behavior and lower levels of physical activity at baseline, raising awareness on specific patient groups that may need special attention in interventions. Besides providing promising intervention targets, our findings may inspire future research to further unravel CVD patients' sitting patterns in relation to individual, environmental, and social factors.

## CONFLICT OF INTEREST

The authors declare that there is no conflict of interest. The results of the present study do not constitute endorsement by ACSM. The results of the present study are presented clearly, honestly, and without fabrication, falsification, or inappropriate data manipulation.

## Supporting information


Appendix S1

## Data Availability

The datasets used and/or analyzed during the current study are stored in a permanent repository and are available from the corresponding author on reasonable request. R code for data processing, analysis, and visualization, are available at OSF (https://osf.io/qjezt/?view_only=323c2b21286f42f08efc5c6b4111e970).

## Footnotes

* For both CVD patients and healthy controls, additional characteristics were measured in the original study, but were not included in the scope of this paper (e.g., education level, marital status). See Bakker et al.^2^ for all measures.

^†^
The difference in average daily sitting time at baseline between CVD patients and healthy controls remained significant after adjustment for potential confounders (i.e., sex, BMI, education level, work status, alcohol use, and smoking status).
