# Supplementary material for: Sitting patterns in cardiovascular disease patients compared with healthy controls and impact of cardiac rehabilitation
Source: Scand J Med Sci Sports. 2022 Jun 18;32(11):1639–49. doi: 10.1111/sms.14202 (PMC9796723; doi:10.1111/sms.14202)
Supplement: Supplementary file 1 — Appendix S1 [file SMS-32-1639-s001.docx]

Supplementary Information for

Sitting patterns in cardiovascular disease patients compared to healthy controls and impact of cardiac rehabilitation

Pam ten Broeke^1^, Bram M. A. van Bakel, Esmée A. Bakker, Debby G. J. Beckers, Sabine A. E. Geurts, Dick H. J. Thijssen^*^, Thijs M. H. Eijsvogels^*^, Erik Bjileveld^*^

^1^ corresponding author

* Shared senior authorship

Email: pam.tenbroeke@ru.nl

Tel: +31 (0) 24 3615729

This Supplementary Information includes:

Supplementary text 1: Details on multilevel time-to-event analysis

Supplementary text 2: Model equations

Supplementary Table 1

Supplementary Table 2

Supplementary Table 3

Supplementary Figure 1

Supplementary Figure 2

**Data-analysis**

***Data preparation for time-to-event analysis***

The event-based summary data file resulting from the activPAL software has a new row for each new activity episode, indicating (a) the start time of the episode, and (b) an activity code, i.e., sitting/lying down, standing, or stepping. We recoded standing and stepping into *active*. We coded a new variable *event*, indicating for each episode the transition that happens at the end of the episode: ‘sit-to-stand’ for each sitting episode, and ‘stand-to-sit’ for each active episode. We then computed *event time* (in minutes; precision in seconds) as the timing of the transition (i.e., event) since the previous transition (i.e., how long a participant had been standing before a stand-to-sit transition; or how long a participant had been sitting before a sit-to-stand transition). For model fitting, we split the dataset based on event of interest: one including only event times for stand-to-sit transitions; one including only event times for sit-to-stand transitions.

***Model fitting***

We performed all statistical analyses in R version 3.6.3. We used the coxph function from the survival package^13^ to fit a separate shared frailty Cox model for each research question (see supplementary text 2 for model equations). The cox approach is well-established for continuous time-to-event data^12^ and is suitable in case no prior knowledge about the shape of the hazard function is available^11,14^. In each model, we included a *frailty term* for participant, accounting for the random variability in baseline hazard between individuals. To compare CVD patients to healthy controls at baseline, we used the pre-CR data of CVD patients and the data of healthy controls. We fitted a shared frailty cox models for each transition (i.e., stand-to-sit; sit-to-stand) with *group* (1 = CVD; 0 = control) as predictor. To explore the relation with time of the day and activity in the preceding 5 hours, we used the pre-CR data of the CVD patients and the data of healthy controls. For each predictor-transition combination, we fitted two shared frailty cox models: Model 1 included the respective predictor and group (1 = CV; 0 = control); in Model 2 we added a Predictor x Group interaction.

To test changes in CVD patients’ sitting from before to after the CR program, we used the pre-CR, post-CR, and follow-up data of the CVD patients. To aid interpretation, age and BMI were standardized on the group level. We fitted a shared frailty cox model for each transition with measurement moment (pre-CR [reference category]; post-CR; follow-up) as predictor. To explore the relation with demographics, disease characteristics, and lifestyle factors, we used the pre-CR, post-CR, and follow-up data of the CVD patients. For each predictor-transition combination, we fitted two shared frailty cox models: Model 1 included the respective predictor and measurement moment (pre-CR; post-CR; follow-up); in Model 2, we added a Predictor x Measurement moment interaction. In all models, we used Efron’s method for handling ties^12^. For all models, the proportionality assumption was met, based on examination of Schoenfeld residuals^12^.

**Model equations**

***Patients versus healthy controls***

The shared frailty Cox model to test the effect of group (1 = CV; 0 = control) as predictor of the hazard of sitting down when standing or the hazard of standing up when sitting was

$$h_{ij}\left( t \right)= h_{0}\left( t \right)\exp\left( v_{i} \right)exp\left( \beta_{1}{group}_{i} \right)$$

In this equation, $h_{ij}\left( t \right)$ is the hazard of sitting down when standing or of standing up when sitting, respectively, for episode *j* within each individual *i*, $h_{0}\left( t \right)$ is the baseline hazard function, $v_{i}$ is the individual-specific random effect (or “frailty”), ${group}_{i}$ is the time-invariant predictor group associated with $\beta_{1}$.

***Time of the day and activity in the preceding 5 hours***

The shared frailty Cox models to test the time-varying predictors time of the day and activity in the preceding 5 hours and the interaction with group as predictors of the hazard of sitting down when standing or the hazard of standing up when sitting were

Model 1: $h_{ij}\left( t \right)= h_{0}\left( t \right)\exp\left( v_{i} \right)exp\left( \beta_{1}X_{ij}\left( t \right)+ \beta_{2}{group}_{i}\left( t \right) \right)$

Model 2: $h_{ij}\left( t \right)= h_{0}\left( t \right)\exp\left( v_{i} \right)exp\left( \beta_{1}X_{ij}\left( t \right) + \beta_{2}{group}_{i}\left( t \right)+\beta_{3}X_{ij}{group}_{i}(t) \right)$

In these equations, $h_{ij}\left( t \right)$ is the hazard of sitting down when standing or of standing up when sitting, respectively, for episode *j* within each individual *i*, $h_{0}\left( t \right)$ is the baseline hazard function, $v_{i}$ is the individual-specific random effect (or “frailty”), $X_{ij}\left( t \right)$ is the time-varying predictor (time of the day or activity in the preceding 5 hours) associated with $\beta_{1}$, ${group}_{i}$ is the time-invariant predictor group associated with $\beta_{2}$, and $X_{ij}{group}_{i}(t)$ is the interaction between the respective predictor and group associated with $\beta_{3}$.

***Changes after a cardiac rehabilitation program***

The shared frailty Cox model to test the time-varying predictors measurement moment (pre-CR [reference category]; post-CR; follow-up) as predictor of the hazard of sitting down when standing or the hazard of standing up when sitting was

$$h_{ij}\left( t \right)= h_{0}\left( t \right)\exp\left( v_{i} \right)exp\left( \beta_{1}{postCR}_{ij}\left( t \right)+ \beta_{2}{followup}_{ij}\left( t \right) \right)$$

In this equation, $h_{ij}\left( t \right)$ is the hazard of sitting down when standing or of standing up when sitting, respectively, for episode *j* within each individual *i*, $h_{0}\left( t \right)$ is the baseline hazard function, $v_{i}$ is the individual-specific random effect (or “frailty”), ${postCR}_{ij}\left( t \right)$ is the time-varying dummy variable comparing post-CR to pre-CR associated with $\beta_{1}$, ${followup}_{ij}\left( t \right)$ is the time-varying dummy variable comparing follow-up to pre-CR associated with $\beta_{2}$.

***Demographics, disease characteristics, and lifestyle factors***

The shared frailty Cox models to test the time-invariant demographics, disease characteristics, and lifestyle factors and the interaction with measurement moment as predictors of the hazard of sitting down when standing or the hazard of standing up when sitting were

Model 1: $h_{ij}\left( t \right)= h_{0}\left( t \right)\exp\left( v_{i} \right)exp\left( \beta_{1}X_{i}\left( t \right)+ \beta_{2}{postCR}_{ij}\left( t \right)+ \beta_{3}{followup}_{ij}\left( t \right) \right)$

Model 2: $h_{ij}\left( t \right)= h_{0}\left( t \right)\exp\left( v_{i} \right)exp\left( \beta_{1}X_{i}\left( t \right)+ \beta_{2}{postCR}_{ij}\left( t \right)+ \beta_{3}{followup}_{ij}\left( t \right)+ \beta_{4}{X_{i}postCR}_{ij}\left( t \right)+ \beta_{5}{X_{i}followup}_{ij}\left( t \right) \right)$

In these equations, $h_{ij}\left( t \right)$ is the hazard of sitting down when standing or of standing up when sitting, respectively, for episode *j* within each individual *i*, $h_{0}\left( t \right)$ is the baseline hazard function, $v_{i}$ is the individual-specific random effect (or “frailty”), $X_{ij}\left( t \right)$ is the time-invariant predictor (demographic characteristic, disease characteristic, or lifestyle factor) associated with $\beta_{1}$, ${postCR}_{ij}\left( t \right)$ is the time-varying dummy variable comparing post-CR to pre-CR associated with $\beta_{2}$, ${followup}_{ij}\left( t \right)$ is the time-varying dummy variable comparing follow-up to pre-CR associated with $\beta_{3}$, ${X_{i}postCR}_{ij}\left( t \right)$ is the interaction between the respective predictor and the dummy variable comparing post-CR to pre-CR associated with $\beta_{4}$, and ${X_{i}followup}_{ij}\left( t \right)$ is the interaction between the respective predictor and the dummy variable comparing follow-up to pre-CR associated with $\beta_{5}$.

**Table S1.** Results of the shared frailty Cox regression models for time of day and the interaction with age, for CVD patients and healthy controls separately.

| **Predictor** | **Estimate** | **df** | **SE** | **HR** | **HR 95% CI** |
| --- | --- | --- | --- | --- | --- |
| CVD patients: Time of day, age, and interaction – hazard of sitting down when standing | | | | | |
| Random effect θ | 0.439*** | 126 |  |  |  |
| Time of day | 0.020*** | 1 | 0.001 | 1.02 | [1.018; 1.023] |
| Age* | -0.048 | 1 | 0.062 | 0.95 | [0.845; 1.075] |
| Time of day x Age | 0.002* | 1 | 0.001 | 1.002 | [1.000; 1.005] |
| Healthy controls: Time of day, age, and interaction – hazard of sitting down when standing | | | | | |
| Random effect θ | 0.278*** | 112.70 |  |  |  |
| Time of day | 0.013 | 1 | 0.001 | 1.01 | [1.011; 1.015] |
| Age | -0.060 | 1 | 0.085 | 0.94 | [0.797; 1.114] |
| Time of day x Age | 0.003** | 1 | 0.001 | 1.003 | [1.001; 1.005] |
| CVD patients: Time of day, age, and interaction – hazard of standing up when sitting | | | | | |
| Random effect θ | 0.330*** | 115.40 |  |  |  |
| Time of day | -0.032*** | 1 | 0.001 | 0.97 | [0.966; 0.970] |
| Age | 0.092 | 1 | 0.183 | 1.10 | [0.766; 1.568] |
| Time of day x Age | -0.007*** | 1 | 0.001 | 0.99 | [0.991; 0.995] |
| Healthy controls: Time of day, age, and interaction – hazard of standing up when sitting | | | | | |
| Random effect θ | 0.333*** | 85.61 |  |  |  |
| Time of day | -0.031*** | 1 | 0.001 | 0.97 | [0.967; 0.971] |
| Age | 0.075 | 1 | 0.291 | 1.08 | [0.609; 1.908] |
| Time of day x Age | -0.005*** | 1 | 0.001 | 0.995 | [0.993; 0.997] |

*** p < .001 ** p < .01 * p < .05

*Note.* df = degrees of freedom, SE = Standard Error, HR = Hazard Ratio, CI = Confidence Interval

**Table S2.** Results of the shared frailty Cox regression models for time of day and activity in the preceding 5 hours at post-CR.

| **Predictor** | **Estimate** | **df** | **SE** | **HR** | **HR 95% CI** |
| --- | --- | --- | --- | --- | --- |
| Time of day, group, and interaction – hazard of standing up when sitting – post CR | | | | | |
| Random effect θ | 0.401*** | 225.2 |  |  |  |
| Time of day | -0.032*** | 1 | 0.001 | 0.97 | [0.967; 0.971] |
| Group | -0.099 | 1 | 0.120 | 0.91 | [0.717; 1.145] |
| Time of day x Group | <0.001 | 1 | 0.002 | 1.00 | [0.997; 1.003] |
| Time of day, group, and interaction – hazard of sitting down when standing – post CR | | | | | |
| Random effect θ | 0.382*** | 226 |  |  |  |
| Time of day | 0.013*** | 1 | 0.001 | 1.01 | [1.011; 1.015] |
| Group | 0.032 | 1 | 0.087 | 1.03 | [0.871; 1.224] |
| Time of day x Group | 0.003* | 1 | 0.002 | 1.003 | [1.000; 1.006] |
| Activity in the preceding 5 hours, group, and interaction – hazard of standing up when sitting – post CR | | | | | |
| Random effect θ | 0.366*** | 225.1 |  |  |  |
| Activity in the preceding 5 hours | -0.009 | 1 | 0.005 | 0.99 | [0.981; 1.001] |
| Group | -0.063 | 1 | 0.110 | 0.94 | [0.758; 1.164] |
| Activity in the preceding 5 hours x Group | -0.023** | 1 | 0.008 | 0.98 | [0.962; 0.994] |
| Activity in the preceding 5 hours, group, and interaction – hazard of sitting down when standing – post CR | | | | | |
| Random effect θ | 0.319*** | 225.6 |  |  |  |
| Activity in the preceding 5 hours | -0.033*** | 1 | 0.005 | 0.97 | [0.958; 0.977] |
| Group | 0.068 | 1 | 0.078 | 1.07 | [0.919; 1.246] |
| Activity in the preceding 5 hours x Group | 0.002 | 1 | 0.008 | 1.002 | [0.987; 1.018] |

*** p < .001 ** p < .01 * p < .05

*Note.* df = degrees of freedom, SE = Standard Error, HR = Hazard Ratio, CI = Confidence Interval

**Table S3.** Results of the shared frailty Cox regression models for time of day and activity in the preceding 5 hours at follow-up.

| **Predictor** | **Estimate** | **df** | **SE** | **HR** | **HR 95% CI** |
| --- | --- | --- | --- | --- | --- |
| Time of day, group, and interaction – hazard of standing up when sitting – follow-up | | | | | |
| Random effect θ | 0.408*** | 223.1 |  |  |  |
| Time of day | -0.032*** | 1 | 0.001 | 0.97 | [0.967; 0.971] |
| Group | -0.063 | 1 | 0.126 | 0.94 | [0.734; 1.201] |
| Time of day x Group | -0.001 | 1 | 0.002 | 0.999 | [0.966; 1.002] |
| Time of day, group, and interaction – hazard of sitting down when standing – follow-up | | | | | |
| Random effect θ | 0.376*** | 223.9 |  |  |  |
| Time of day | 0.013*** | 1 | 0.001 | 1.01 | [1.011; 1.015] |
| Group | -0.013 | 1 | 0.091 | 0.99 | [0.826; 1.181] |
| Time of day x Group | 0.007*** | 1 | 0.002 | 1.01 | [1.004; 1.010] |
| Activity in the preceding 5 hours, group, and interaction – hazard of standing up when sitting – follow-up | | | | | |
| Random effect θ | 0.354*** | 223.1 |  |  |  |
| Activity in the preceding 5 hours | -0.009 | 1 | 0.005 | 0.99 | [0.981; 1.001] |
| Group | -0.078 | 1 | 0.109 | 0.93 | [0.747; 1.146] |
| Activity in the preceding 5 hours x Group | -0.008 | 1 | 0.008 | 0.99 | [0.976; 1.008] |
| Activity in the preceding 5 hours, group, and interaction – hazard of sitting down when standing – follow-up | | | | | |
| Random effect θ | 0.304*** | 223.5 |  |  |  |
| Activity in the preceding 5 hours | -0.033*** | 1 | 0.005 | 0.97 | [0.959; 0.977] |
| Group | 0.079 | 1 | 0.080 | 1.08 | [0.925; 1.267] |
| Activity in the preceding 5 hours x Group | 0.003 | 1 | 0.008 | 1.003 | [0.988; 1.018] |

*** p < .001

*Note.* df = degrees of freedom, SE = Standard Error, HR = Hazard Ratio, CI = Confidence Interval


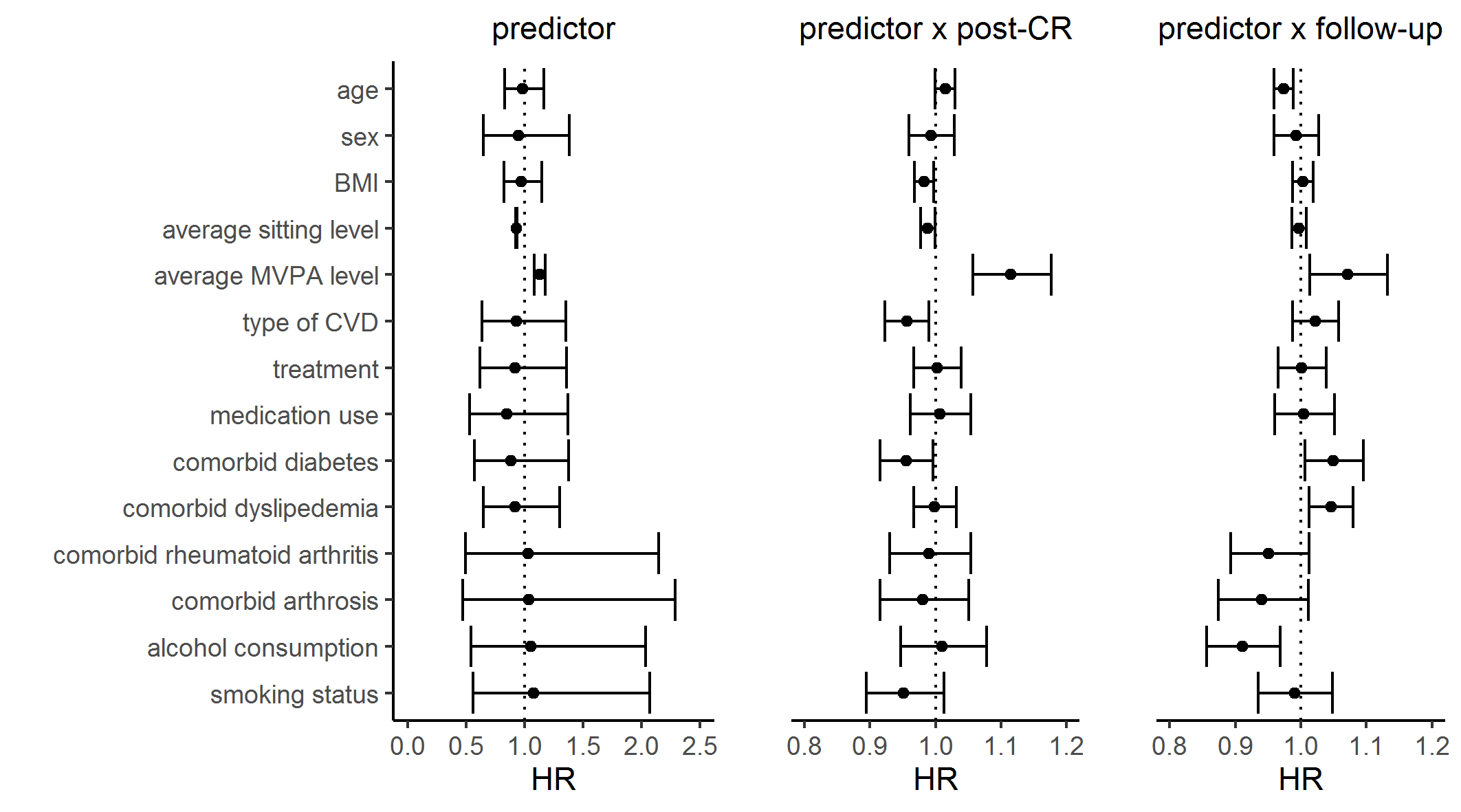


**Figure S1.** Results of the shared frailty Cox regression models predicting the hazard of standing up when sitting with demographics, patient characteristics, or lifestyle factor, and the interaction with measurement moment. The figure shows the Hazard Ratio (HR) and accompanying confidence interval (error bars) for the main effect of the predictor of interest (left panel), the interaction with the dummy-variable comparing post-CR to pre-CR (middle panel) or the interaction with the dummy-variable comparing follow-up to pre-CR (right panel).

The figure shows that average sitting level negatively predicts the hazard of standing up when sitting, and average MVPA level positively predicts of the hazard of standing up when sitting. Also, older age, higher BMI, and higher sitting level were associated with a *weaker increase* in the hazard of standing up when sitting, as indicated by a HR < 1 for the interaction with measurement moment. Higher MVPA level was associated with a *stronger increase* in the hazard of standing up when sitting, as indicated by a HR > 1 for the interaction with measurement moment.


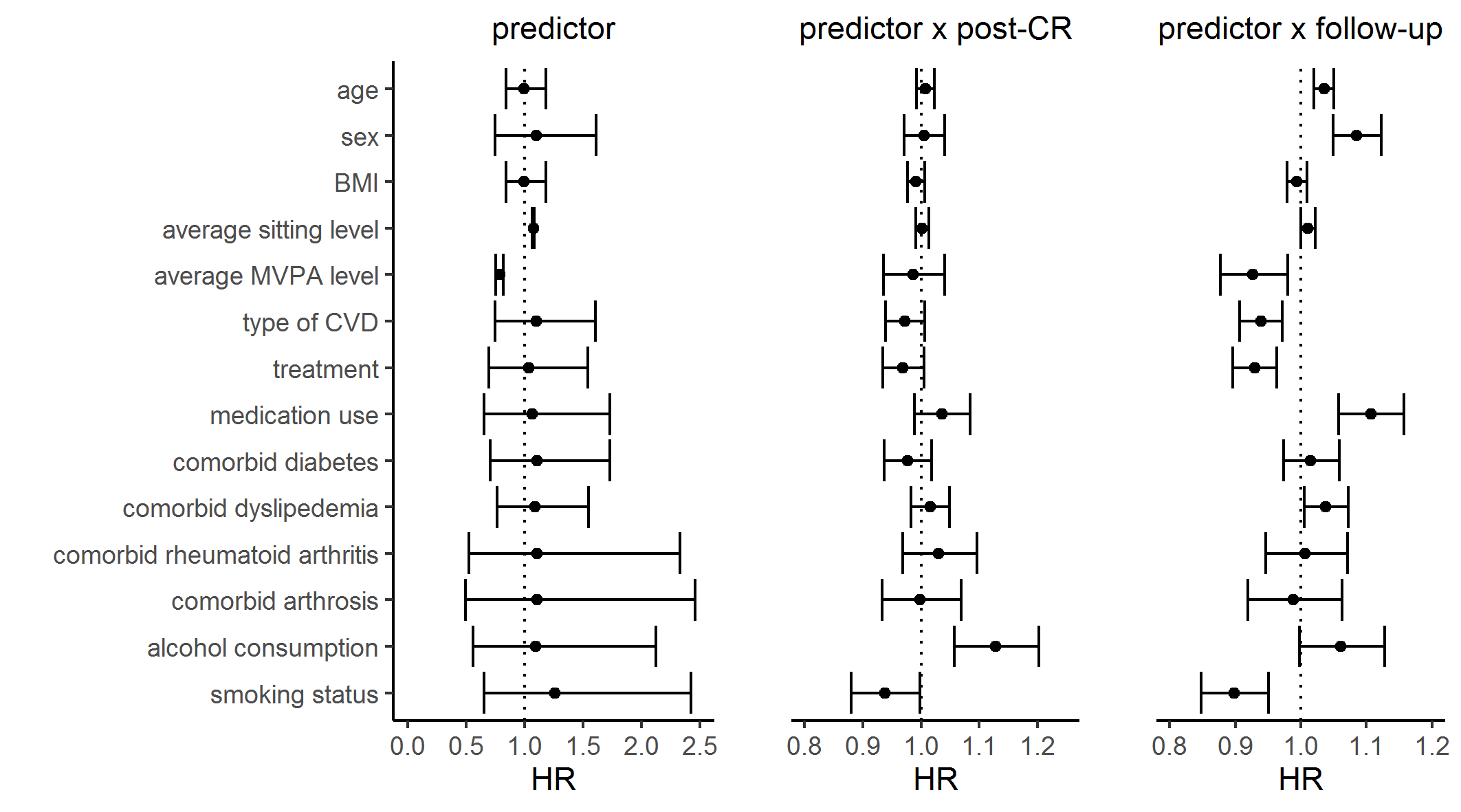


**Figure S2.** Results of the shared frailty Cox regression models predicting the hazard of sitting down when standing with demographics, patient characteristics, or lifestyle factors, and the interaction with measurement moment. The figure shows the Hazard Ratio (HR) and accompanying confidence interval (error bars) for the main effect of the predictor of interest (left panel), the interaction with the dummy-variable comparing post-CR to pre-CR (middle panel) or the interaction with the dummy-variable comparing follow-up to pre-CR (right panel).

The figure shows that average sitting level positively predicts the hazard of sitting down when standing, and average MVPA level negatively predicts the hazard of sitting down when standing. Older age and higher sitting level were associated with a *weaker decrease* in the hazard of sitting down when standing, as indicated by a HR > 1 for the interaction with measurement moment. Higher MVPA level was associated with a *stronger decrease* in the hazard of sitting down when standing, as indicated by a HR < 1 for the interaction with measurement moment.
